# Factors Affecting the Weaning from Nasal CPAP in Preterm Neonates

**DOI:** 10.1155/2012/416073

**Published:** 2011-12-08

**Authors:** Shantanu Rastogi, Hariprem Rajasekhar, Anju Gupta, Alok Bhutada, Deepa Rastogi, Jen-Tien Wung

**Affiliations:** ^1^Division of Neonatology, Maimonides Infants Children Hospital of Brooklyn, 1048 Tenth Avenue, G-103, Brooklyn, NY 11203, USA; ^2^SUNY Health Science Center at Brooklyn, NY 11203-2098, USA; ^3^Division of Respiratory and Sleep Medicine, Children's Hospital at Montefiore, Albert Einstein College of Medicine, 3415 Bainbridge Avenue, Bronx, NY 10467, USA; ^4^Division of Neonatology, Morgan Stanley Children's Hospital of New York, College of Physicians and Surgeons, Columbia University, Broadway, NY 10032, USA

## Abstract

*Objective*. Identification of the weight and postmenstrual age (PMA) at successful weaning of NCPAP in preterm neonates and the factors influencing the successful wean. *Study Design*. Retrospective review of 454 neonates ≤32 weeks of gestational age (GA) who were placed on NCPAP and successfully weaned to room air was performed. *Results*. Neonates had a mean birth weight (BW) of 1357 ± 392 grams with a mean GA of 29.3 ± 2.2 weeks. Neonates were weaned off NCPAP at mean weight of 1611 ± 432 grams and mean PMA of 32.9 ± 2.4 weeks. Univariate analysis showed that chorioamnionitis, intubation, surfactant use, PDA, sepsis/NEC, anemia, apnea, GER and IVH were significantly associated with the time to NCPAP wean. On multivariate analysis, among neonates that were intubated, BW was the only significant factor (*P* < 0.001) that was inversely related to time to successful NCPAP wean. Amongst non-intubated neonates, along with BW (*P* < 0.01), chorioamnionitis (*P* < 0.01), anemia (*P* < 0.0001), and GER (*P* < 0.02) played a significant role in weaning from NCPAP. *Conclusion*. Neonates were weaned off NCPAP at mean weight of 1611 ± 432 grams and mean PMA of 32.9 ± 2.4 weeks. BW significantly affects weaning among intubated and non-intubated neonates, though in neonates who were never intubated chorioamnionitis, anemia and GER also significantly affected the duration on NCPAP.

## 1. Introduction

Treatment of neonatal respiratory distress syndrome (RDS) with intermittent positive pressure ventilation (IPPV) has been associated with significant pulmonary morbidity. Studies have shown that this morbidity can be reduced by use of nasal continuous positive airway pressure (NCPAP) [1–4], leading to increased use of NCPAP for the management of RDS in preterm neonates in recent years. Multiple studies have also demonstrated that NCPAP is a safe treatment modality with no increase in short-term [5–10] and long-term [11, 12] morbidities. It also has other beneficial effects including induction of lung growth [13]. Although NCPAP has been used more routinely, there is paucity of information on factors affecting the weaning process such as weight and PMA at the time of successful weaning and the factors that influence the weaning process [14, 15].

Recently, studies have been conducted to evaluate the methods of NCPAP wean used at several neonatal intensive care units (NICUs). These identified a lack of agreement on the method of NCPAP wean used at various NICUs with only 6% of the participating NICUs having written guidelines regarding NCPAP wean. The start of the NCPAP weaning was frequently arbitrarily determined by healthcare providers (physicians, nurses, and respiratory technicians). There was variability in the method of weaning from NCPAP, which was attempted either by gradually increasing the time of NCPAP, by reducing pressure, or by using both methods [16, 17]. Additionally, these surveys did not identify the age, weight, or associated clinical comorbidities, which may affect successful weaning from NCPAP in preterm neonates. A recent Cochrane review on weaning from NCPAP in preterm neonates also highlighted the lack of information available on the weaning from NCPAP [18].

Given this paucity of information, the objective of our study was to identify the PMA and weight at which one can successfully wean preterm neonates born ≤32 weeks of gestational age off the NCPAP and the comorbidities that may influence the success of NCPAP weaning.

## 2. Materials and Methods

### 2.1. Patients

A retrospective chart review was conducted on all babies born at GA of ≤32 weeks who were admitted to the NICU at Maimonides Infants and Children's Hospital, Brooklyn, between 1st of January 2003 and 31st of December 2007. This study was approved by the institutional review board at Maimonides Medical Center and was conducted in compliance with Health Insurance Portability and Accountability Act regulations.

There were 648 babies who were born at ≤32 weeks of GA and admitted to the NICU during the study period. We excluded all neonates who were stable in room air and did not need any respiratory support (*n* = 103) and those who died or transferred out while intubated or before they could be weaned off NCPAP (*n* = 85). Those who were placed on nasal cannula before meeting the criteria of successful weaning from NCPAP (*n* = 6) were also excluded. Nasal SIMV was not used in our nursery during the study period. Thus, data from 454 eligible neonates was analyzed for the study.

The primary variables studied were BW, GA, ethnicity, and gender. Additionally, weights and the PMA at the following four time points were obtained: (1) when the neonates were placed on NCPAP, (2) when they reached FiO_2_ of 0.21 on NCPAP, (3) when weaning from NCPAP was initiated, and (4) when the NCPAP was successfully weaned off. The association of antenatal factors and postnatal co-morbidities with the time to NCPAP weaning was also studied. Antenatal factors such as use of antenatal steroids (complete course) and magnesium sulphate, presence of chorioamnionitis, preeclampsia (blood pressure of more than 140/90 with proteinuria), and intrauterine growth retardation (IUGR) (less than 3rd percentile on the growth curve) were analyzed. Postnatal factors included in the analysis were intubation prior to weaning from NCPAP, use of surfactant, the presence of patent ductus arteriosus (PDA), diagnosed in first week (confirmed by echocardiography), anemia (hematocrit of <30 1 week prior to the initiation of NCPAP weaning), GER (diagnosed clinically and treated with H_2_ blocker or proton pump inhibitor), apnea (cessation of respiration for >20 seconds associated with bradycardia or cyanosis) >2 in 12 hours or >3 in 24 hours with, at least, one requiring bag and mask ventilation, and presence of intra-ventricular hemorrhage (IVH) (diagnosed by ultrasound). In addition, occurrence of sepsis/necrotizing enterocolitis (NEC) (culture positive or radiologically proven) was also included in the analysis. These two clinical conditions were analyzed together as both similarly affect the respiratory system and hence the duration time on NCPAP through the effect of inflammatory mediators on the lungs. Caffeine was exclusively used to treat neonates diagnosed with apnea.

### 2.2. Respiratory Management

A uniform method of respiratory management has been practiced in our NICU [19, 20] for over a decade. In summary, all spontaneously breathing neonates with respiratory distress are placed on bubble NCPAP using the Hudson RCI nasal prongs (Hudson Respiratory Care, Temecula, California, USA) with 5 cm H_2_O pressure within first 10 minutes of life irrespective of GA and BW of the neonate. Neonates who are not breathing spontaneously at birth or fail a trial of NCPAP are intubated. Failure of NCPAP is defined as increased work of breathing determined clinically by persistent tachypnea (>60/minute for >2 hours) and marked retractions, apnea as defined above, abnormal blood gases (2 arterial samples >2 hours apart) low pH < 7.2, PaCO_2_ > 65 mm of Hg, and PaO_2_= of <50 mm of Hg with FiO_2_ of ≥0.4. Surfactant is only used as rescue treatment. The peak end expiratory pressure is kept at 5 cm H_2_O during the weaning process.

NCPAP weaning is initiated when the neonate is clinically stable on room air NCPAP for 48 hours. When neonates are weaned off NCPAP, special attention is paid to upper airway suctioning and to keeping the neck in a neutral position to prevent excessive flexion or extension. Success of weaning from NCPAP is defined as the baby being stable in room air without any respiratory support for 7 days.

### 2.3. Statistical Analysis

Statistical analysis was done using STATA version 10 (StataCorp LP. College Station, Tex, USA). Continuous variables such as BW, GA, PMA at various time points of NCPAP weaning, and hematocrits were evaluated for normality. While BW was normally distributed, GA was not. As expected, those born at a younger GA were on NCPAP for a longer duration. To accommodate to this, we used PMA at complete weaning of NCPAP as the outcome variable of interest, since this was normally distributed in our sample. Univariate analysis was conducted using the *t*-test or analysis of variance for continuous variables and chi-square test for categorical variables to identify the association of clinical variables with the PMA at NCPAP weaning. To study the association of ethnicity with NCPAP weaning, Caucasians were used as the reference group. The Spearman correlation was used to study the association of NCPAP weaning with continuous variables such as hematocrit levels.

 Linear regression analysis was done to identify the significance of the association of the variables identified by univariate analysis on the primary outcome variable of interest, that is, the PMA at successful weaning from NCPAP when adjusted for other clinical variables. Variables of epidemiological significance such as gender were retained in the model even though they did not reach statistical significance in univariate analysis. We identified a significant interaction between birth weight and intubation. To account for this, a stratified analysis was performed, based on intubation status, as shown in Table 4. In addition, there was significant collinearity between intubation and surfactant, PDA, and apnea. For this reason, these variables were not included in the final stratified model. Regression diagnostics were performed to ensure that assumptions of linear regression analysis were not violated. 

## 3. Results

For the entire study population, the mean BW was 1357 ± 392 grams and mean GA was 29.3 ± 2.2 weeks. Demographic and clinical data of the study cohort as a whole and when stratified by intubation is shown in Table 1. There was no effect of gender or of ethnicity on the PMA at successful NCPAP weaning. 

The weights and PMA of the neonates at various NCPAP time points are shown in Table 2. The mean GA of those requiring intubation was 27.3 ± 2.1 weeks and of those not requiring intubation was 30.1 ± 1.7 weeks (*P* < 0.0001). There were significant differences between those who were intubated and those who did not require intubation in the PMA and their weights at various NCPAP time points. 

**Figure 1:**
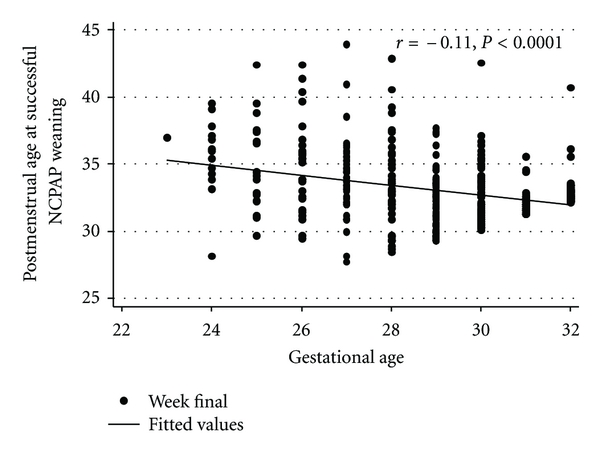
Correlation between GA and PMA at successful NCPAP weaning.

**Figure 2:**
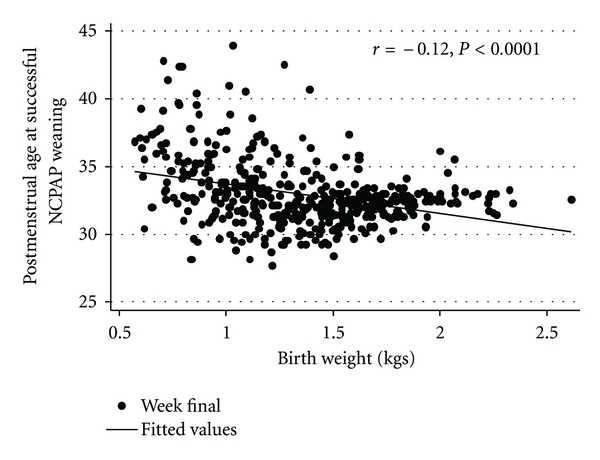
Correlation between BW and PMA at successful NCPAP weaning.

As shown in Figures 1 and 2, there was a significant inverse correlation between GA (*r* = −0.11, *P* < 0.0001) and BW (*r* = −0.12, *P* < 0.0001) to PMA at successful NCPAP weaning. The beta for the regression line for GA was −0.4; hence, the duration on NCPAP decreased by 0.4 weeks for every week increase in GA. Similarly the beta for the regression line for BW was −0.21; the duration of NCPAP decreased by 0.2 weeks for every 100 grams increase in BW.

 Univariate analysis of clinical comorbidities with PMA at successful NCPAP weaning is shown in Table 3. There was a significant association of PMA at successful NCPAP weaning with chorioamnionitis, intubation, and comorbidities such as PDA, sepsis/NEC, anemia, apnea, GER, and IVH. There was significant association between PDA, apnea, and surfactant use and successful NCPAP weaning in our study, but since there was collinearity between these variables and intubation, we used intubation as a marker for these variables in multivariate analysis. In addition to BW and GA, successful weaning had a significant inverse correlation with hematocrit (*r* = −0.44, *P* < 0.001) and positive correlation with the severity of IVH (*r* = 0.34, *P* < 0.0001).

As shown in Table 4, in multivariate analysis, BW, chorioamnionitis, anemia, GER, and IVH were independent predictors for successful NCPAP weaning. In the stratified model, among those neonates who were not intubated, BW, chorioamnionitis, anemia, and GER remained independent predictors of successful NCPAP weaning. On the other hand, among those who were intubated prior to weaning, BW was the only independent predictor of successful NCPAP weaning with the other comorbid conditions not reaching statistical significance. In the whole cohort, length of stay on NCPAP increased by 1.1 weeks in those who had chorioamnionitis, 1 week in those who had anemia, and 1.9 weeks in those who had GER.

## 4. Discussion

We found that GA and BW were strongly correlated with the duration of NCPAP in neonates born less than 32 weeks of gestation but there was no association with ethnicity or gender. These findings differ from previous studies that have demonstrated links between ethnicity and the severity of RDS [21]. These differences in the observation may be due to the relatively small sample size of our study as gender- and ethnicity-specific difference in disease severity have been elucidated in large-population-based epidemiological studies.

While several factors were found to be associated with increased time on NCPAP, we identified that there was a difference in their role among neonates that were intubated and those who were not intubated. The time to successful NCPAP weaning was longer among nonintubated preterm neonates who had evidence of maternal chorioamnionitis, anemia, and gastroesophageal reflux. However, among those neonates who were intubated, weaning from NCPAP was not associated with any of these factors.

In the nonintubated neonates, maternal chorioamnionitis was associated with increased length of time on NCPAP, which may be due to the injury caused by inflammatory mediators to the immature lungs, predisposing them to develop chronic lung disease [22–24]. The effects of anemia on the duration of NCPAP are likely due to decrease in oxygen delivery and increase in cardiac load and work of breathing. Studies have linked anemia with failure of extubation in children and adults, but similar findings have not been reported with weaning of NCPAP [25]. The effect of GER on failure to wean off NCPAP could be related to its association with increased incidence and severity of apneas related to acid reflux and associated lung inflammation related to frequent aspirations [26]. However, prior studies have failed to demonstrate this association [27]. We also found preterm neonates with GER had a higher incidence of BPD. This has been previously reported and may further explain the association between GER and the longer duration of NCPAP use [28]. While we have identified these associations, further studies are needed to corroborate these findings since they have direct clinical application and significance on management of preterm neonates. However, the associations identified in our study highlight the need for appropriate identification and management of these comorbid conditions to facilitate early weaning of NCPAP.

The delayed weaning from NCPAP in the intubated preterm neonates not only may be related to the immaturity of the lungs (as the intubated preterm neonates had lower gestational age), but could also be due to the ventilator-induced lung injury associated with intubation and positive-pressure ventilation. The process of intubation not only increases the chances of hemodynamic instability [29] but also bridges the unsterile upper airway to that of the lower airway leading to increased incidence of colonization and infection of the lower airways and lungs causing lung injury [30]. Positive-pressure ventilation also causes trauma to the lungs by multiple mechanisms such as barotrauma, volutrauma, atelectrauma, and biotrauma [31]. In multivariate analysis, BW was the only significant factor determining time for weaning among those preterm neonates who were intubated with no association identified with the comorbid conditions that were found to be significant in those who were not intubated. It may be hypothesized that the role of these comorbidities was masked by the immaturity of the lungs and severity of ventilation-induced injury in the intubated neonates. Furthermore, intubated neonates were on NCPAP with FiO_2_ of 0.21 longer than their nonintubated counterparts. As intubation and ventilation are associated with pulmonary injury and NCPAP helps in lung growth and repair [13], intubated neonates took longer to reach a point at which they could be weaned off NCPAP even when they reached room air NCPAP. However this is the first study to identify differences in the association of clinical morbidities with NCPAP weaning among neonates by intubation status. Hence future studies are needed to corroborate our findings.

We recognize that caffeine use was limited to the preterm neonates with significant apnea. These neonates were on NCPAP for a longer duration than those without apneas. In keeping with the findings of Schmidt et.al. [32], if all our preterm neonates had received caffeine, the difference between the duration of NCPAP in the nonintubated and the intubated neonates would have been expected to more pronounced than that observed, as the nonintubated neonates would be able to come off NCPAP even earlier.

There are certain limitations to this study, specifically, those inherent to retrospective analysis and those related to the information derived from a single center. One feature which is unique to our unit is the lower incidence of bronchopulmonary dysplasia (BPD), despite a similar incidence of RDS, compared to other centers in the Vermont Oxford database. The average incidence of BPD in less than 1500 gram preterm neonates in our NICU varied from 7.4% to 10.5% as compared to the 26.7% to 29.7% in the database during the 5-year study period. However, as NCPAP has been associated with lower incidence of BPD rates [33], we attribute these differences to the higher use of NCPAP in our NICU as compared to other centers. Further, the diagnosis of GER was made clinically and not by pH probe, but if a pH probe study was performed, more cases of subclinical GER would be diagnosed likely making the association of weaning failure and GER even stronger. Moreover, since anemia was significantly related to the success of NCPAP weaning, transfusions may have impacted the success of weaning. However, as the transfusion threshold was the same for all the neonates prior to the weaning of NCPAP, irrespective of the intubation status, it was likely not associated with any misclassification bias. Additionally, we did not use nasal SIMV during NCPAP weaning. We recognize that the judicious use of this technique might have reduced the need of ventilation and may have decreased the time on NCPAP as suggested by a recent review [34]. Another limitation in this study was the inability to determine which method of weaning (sudden removal of NCPAP, gradual weaning by decreasing pressure or by gradually increasing time off NCPAP) was better in getting the neonates weaned from the NCPAP earlier or if any factors studied had different relationship with the weaning techniques. This was difficult to evaluate due to the retrospective nature of the study. Further studies are needed to evaluate the best method to wean preterm neonates off NCPAP.

In conclusion, our findings suggest that in preterm neonates, NCPAP can be successfully weaned off at a mean PMA of 32.9 ± 2.4 weeks and weight of 1611 ± 432 grams. There was an inverse relationship between time to successful NCPAP weaning and BW. In addition, among those neonates who were not intubated, prevention of maternal chorioamnionitis, identification and treatment of anemia and GER may reduce the time on NCPAP. Our findings provide neonatal health care providers clinical information on successful NCPAP weaning that may be used to initiate wean and help in decreasing the number of weaning failures. This information could also be used to counsel parents on the time when neonates could be expected to be successfully weaned from NCPAP. As our findings are from a single clinical center, future multicenter trials are needed to study whether these factors uniformly influence NCPAP wean in different neonatal intensive care units.

## Figures and Tables

**Table 1:** Demographic and clinical data of the study population.

Demographic/clinical characteristic	Full cohort *n* = 454	Nonintubated *n* = 326	Intubated *n* = 128	*P* value
	*n* (%)	*n* (%)	*n* (%)	
Male	242 (53.3)	179 (54.9)	63 (49.2)	0.29
*Ethnicity*				
White	133 (29.3)	93 (28.5)	40 (31.3)	0.59
African Americans	79 (17.4)	58 (17.8)	21 (16.4)	
Hispanics	108 (23.8)	83 (25.5)	25 (19.5)	
Asians	88 (19.4)	63 (19.1)	26 (20.3)	
Multiracial	46 (10.1)	30 (9.2)	16 (12.5)	
Antenatal steroids	361(79.5)	257 (78.8)	104 (81.3)	0.56
Chorioamnionitis	21 (4.6)	14 (4.5)	7 (5.5)	0.58
Preeclampsia	72 (15.9)	54 (16.6)	18 (14.1)	0.50
Magnesium sulphate use	203 (44.7)	135 (41.1)	68 (53.1)	0.02
IUGR	19 (4.2)	13 (3.9)	6 (4.7)	0.74
Intubation	128 (28.3)			
Surfactant	89 (19.6)	0 (0)	89 (69.5)	
IVH	69 (15.1)	26 (8.0)	43 (33.6)	<0.001
PDA	167 (36.9)	19 (5.8)	66 (51.6)	<0.0001
Sepsis/NEC	40 (8.8)	20 (6.1)	20 (15.6)	<0.001
Anemia	273 (80.1)	150 (46.0)	123 (96.0)	<0.0001
Apnea	100 (22.3)	45 (13.8)	55 (42.9)	<0.0001
GE reflux	29 (6.9)	7 (2.1)	22 (17.2)	<0.001
BPD	50	7 (2.1)	43 (33.6)	<0.001

Abbreviations: IUGR: intrauterine growth retardation, PDA: patent ductus arteriosus, IVH: intraventricular hemorhage, NEC: necrotizing enterocolitis, GE reflux: gastroesophageal reflux.

**Table 2:** Weight (grams) and postmenstrual age (PMA in weeks) at various NCPAP time points, for the entire cohort and for subgroups stratified by intubation.

	Full cohort (*n* = 454)		Nonintubated (*n* = 326)		Intubation (*n* = 128)	
	Weight	PMA	Weight	PMA	Weight	PMA
At start of NCPAP	1356 ± 392	29.6 ± 2.1	1480 ± 346	30.3 ± 1.7	1037 ± 315*	27.9 ± 2.1***
Reaching 0.21 FiO_2_ NCPAP	1342 ± 377	30.1 ± 2.1	1441 ± 344	30.5 ± 1.7	1089 ± 339*	29 ± 2.6*
Initiation of NCPAP weaning	1492 ± 376	31.9 ± 2.2	1462 ± 345	31.3 ± 1.4	1570 ± 438*	33.2 ± 2.9*
Successful NCPAP weaning	1611 ± 432	32.9 ± 2.5	1580 ± 381	32.1 ± 1.7	1869 ± 484**	35 ± 2.9**

*P < 0.01, **P < 0.001, ***P < 0.0001 when the weights and PMA of intubated neonates were compared to those nonintubated.

**Table 3:** Effect of clinical factors on PMA in weeks at successful NCPAP weaning.

	Present*	Absent*	*P*-value
Antenatal steroids	32.9±2.5	32.7±2.2	*P* = 0.38
Chorioamnionitis	34.1±2.8	32.9±2.4	*P* < 0.02
Preeclampsia	32.5±2.2	33±2.5	*P* = 0.14
Magnesium sulphate use	33.1±2.7	32.8±2.2	*P* = 0.26
IUGR	33.1±2.2	32.9± 2.5	*P* = 0.72
Intubation	35.0±2.9	32.1±1.7	*P* < 0.00001
PDA	34.2±2.9	32.2±1.7	*P* < 0.0001
Sepsis/NEC	34.2± 2.5	32.8±2.4	*P* < 0.0001
Anemia	33.7±2.8	31.8±1.2	*P* < 0.0001
Apnea	33.9±2.6	32.7±2.3	*P* < 0.0001
GE reflux	35.6± 3.5	32.8±2.3	*P* < 0.00001
IVH (grades3/4)	34.5±3.4	32.6±2.1	*P* < 0.0001

* PMA is reported as mean ±SD.

Abbreviations: IUGR: intrauterine growth retardation, PDA: patent ductus arteriosus, NEC: necrotizing enterocolitis, GE Reflux: gastroesophageal reflux, IVH: intraventricular hemorhage.

**Table 4:** Multivariate analysis for PMA at successful NCPAP weaning and significant predictor variables, analysis done for the group as a whole and when stratified by intubation.

	Full cohort (*n* = 454)			Nonintubated (*n* = 326)			Intubated (*n* = 128)		
	Beta	95% CI	*P* value	Beta	95% CI	*P* value	Beta	95% CI	*P* value
BW	−0.90	−1.50–0.28	0.004	−0.76	−0.17–1.30	0.01	−2.50	−4.02–0.99	0.001
Gender	−0.14	−0.54–0.26	0.5	−0.15	−0.51–0.20	0.39	−0.13	−1.10–0.85	0.8
Chorioamnionitis	1.14	0.19–2.10	<0.01	0.72	0.16–1.60	0.01	0.95	−1.18–3.08	0.38
Sepsis/NEC	0.67	−0.05–1.38	0.06	0.71	−0.03–1.50	0.06	0.21	−1.12–1.54	0.75
Anemia	1.05	0.56–1.55	<.0001	0.91	0.50–1.30	<.001	0.61	−2.10–3.40	0.69
IVH	1.14	0.57–1.72	<.01	0.52	−0.13–1.18	0.1	0.80	−0.20–1.82	0.12
GE reflux	1.99	1.16–2.82	<.0001	1.44	0.23–2.65	.02	1.23	−0.06–2.52	0.06

Abbreviations: BW: birth weight, IVH: intraventricular hemmorhage, NEC: necrotizing enterocolitis, GE Reflux: gastroesophageal reflux.

## Abbreviations

PMA: Postmenstrual age
GA: Gestational age
BW: Birth weight
NCPAP: Nasal continuous positive airway pressure
GER: Gastro-esophageal reflux
RDS: Respiratory distress syndrome
IPPV: Intermittent positive pressure ventilation
NICU: Neonatal intensive care unit
IUGR: Intra-uterine growth retardation
PDA: Patent ductus arteriosus
NEC: Necrotizing enterocolitis
IVH: Intraventricular hemorrhage
BPD: Broncho-pulmonary dysplasia.
